# Exploring the Impact of Language Services on Utilization and Clinical Outcomes for Diabetics

**DOI:** 10.1371/journal.pone.0038507

**Published:** 2012-06-04

**Authors:** Karen Hacker, Yoon Susan Choi, Lisa Trebino, LeRoi Hicks, Elisa Friedman, Bonnie Blanchfield, G. Scott Gazelle

**Affiliations:** 1 Institute for Community Health, Cambridge Health Alliance, Cambridge, Massachusetts, United States of America; 2 Harvard Medical School, Boston, Massachusetts, United States of America; 3 Umass Memorial Health Care, University of Massachusetts Medical School, Worcester, Massachusetts, United States of America; 4 Institute for Technology Assessment, Massachusetts General Hospital, Boston, Massachusetts, United States of America; Yale University School of Medicine, United States of America

## Abstract

**Background:**

Significant health disparities exist between limited English proficient and English-proficient patients. Little is known about the impact of language services on chronic disease outcomes such as for diabetes.

**Methods/Principal Findings:**

To determine whether the amount and type of language services received during primary care visits had an impact on diabetes-related outcomes (hospitalization, emergency room utilization, glycemic control) in limited English proficient patients, a retrospective cohort design was utilized. Hospital and medical record data was examined for 1425 limited English proficient patients in the Cambridge Health Alliance diabetes registry. We categorized patients receiving usual care into 7 groups based on the amount and combination of language services (language concordant providers, formal interpretation and nothing) received at primary care visits during a 9 month period. Bivariate analyses and multiple logistic regression were used to determine relationships between language service categories and outcomes in the subsequent 6 months. Thirty-one percent of patients (445) had no documentation of interpreter use or seeing a language concordant provider in any visits. Patients who received 100% of their primary care visits with language concordant providers were least likely to have diabetes-related emergency department visits compared to other groups (p<0001) in the following 6 months. Patients with higher numbers of co-morbidities were more likely to receive formal interpretation.

**Conclusions/Significance:**

Language concordant providers may help reduce health care utilization for limited English proficient patients with diabetes. However, given the lack of such providers in sufficient numbers to meet patients' communication needs, strategies are needed to both increase their numbers and ensure that the highest risk patients receive the most appropriate language services. In addition, systems serving diverse populations must clarify why some limited English proficient patients do not receive language services at some or all of their visits and whether this has an impact on quality of care.

## Introduction

The impact of language barriers on the delivery of health services to limited English proficient patients has been well established [1]. Significant health disparities exist for limited English proficient patients who are less likely to receive preventive services [2], [3] and health education, [4], [5] and more likely to lack continuity of care and experience problems understanding medical recommendations than English-proficient patients [6], [7], [8]. Language barriers have been implicated in reduced medication adherence [9], greater likelihood of hospital admission, [10] longer hospital stays [11], and increased resource utilization [12].

Competent language assistance has been shown to lower barriers by increasing access to and quality of care [13], [14]. Language services provided by professional medical interpreters or bilingual staff/providers have been associated with various positive outcomes for limited English proficient patients, including increased preventive screening rates, [15], [16] greater likelihood of receiving lifestyle counseling [17], greater satisfaction with care, [18], [19] increased treatment compliance, [20] and reduced emergency department return rates [21].

While studies have focused on the experience of care and elements of the care process, little is known about how language services impact specific disease outcomes in limited English proficient patients [13], [22], [23], [24]. Diabetes serves as a prime example of a disease whose clinical outcomes may be influenced by the use of language services given the inherent communication demands in care (i.e., ongoing self management education and frequent interaction with the healthcare system) [25], [26], [27]. Yet, studies examining the impact of language services on diabetes related outcomes offer conflicting results [28], [29], [30].

Prior researchers have also been limited in their ability to compare the effects of either *increasing* or *decreasing* language services to limited English proficient patients, resulting in studies that generally compare effectiveness of various language service modalities (trained medical interpreters, untrained family members or clinical staff, and language concordant providers) [28], [31], [32], [33], [34]. While these studies have found differences between the modalities, additional real world studies are needed to elucidate the realities of language service utilization as well as the impact of these services on chronic care outcomes.

To address the limitations of prior literature, we examined clinical data on diabetic limited English proficient patients from a public health safety-net system to determine the impact of modality and amount of language services received at primary care visits on clinical outcomes related to diabetes (hospitalization, emergency room utilization, glycemic control). In addition, we aimed to explore the modality and amount of language services received by diabetic limited English proficient patients over the study period.

## Methods

### Ethics Statement

The Cambridge Health Alliance Institutional Review Board Approval for the study was received on June 9, 2008. No consent was needed as data was analyzed anonymously and the committee waived the need for consent.

### Study Setting

The study setting was the Cambridge Health Alliance, an integrated public health system in Massachusetts. Cambridge Health Alliance cares for a diverse and largely immigrant population, a third of whom speak a primary language other than English [35]. Language services are available via formal interpretation as well as language-concordant providers. Formal interpretation is available in 75 languages and provided by trained medical interpreters either face-to-face or via telephone. While no industry-wide requirement exists, Cambridge Health Alliance requires that all interpreters (including telephonic interpreters) complete 80 hours of medical interpreter training and pass an independent interpreter skills assessment provided by a third party. Ninety percent of all face-to-face and telephonic interpreting is provided by Cambridge Health Alliance in-house interpreters and the remaining 10% is provided by the Language Line, a language service phone line, which employs similarly trained interpreters.

It is standard policy at Cambridge Health Alliance that any patient with limited English proficiency should have access to an interpreter. At the point of registration at Cambridge Health Alliance, patients are asked the following questions: “What is your primary language?” In which language do you prefer to communicate with your health care provider?” and “Would you like to use interpreter services for your visit?” For both language questions patients can only give one response. Staff asks these questions at the initial registration of a patient and if the patient prefers a language other than English, they will ask the patient about their interpreter needs when an appointment is scheduled and again at the appointment. Providers can also assess the need for an interpreter during an encounter and request one. All registration staff and providers are trained both on accessing interpreter services and on the importance of and rationale for asking about patient language preference. In addition, all new employees receive a 45 minute training session on providing language and culturally appropriate services as part of orientation, and all employees are required to complete an annual test on Cambridge Health Alliance policies which includes a section on how to ask patients about their language needs and how to access interpreter services. Specific training is also available by request from clinic leadership or when issues arise.

In addition to their interpreter workforce, Cambridge Health Alliance employs many providers who are multi-lingual. At the time of this study, there were over 80 language-concordant providers in the system who collectively spoke approximately 20 languages. A provider who speaks the same language as the patient (language-concordant provider) can be requested when making an appointment but not all requests can be accommodated. Typically providers identify their language fluency as part of the hiring process, and this is uploaded into the provider registry. There is no formal means (including a standard set of questions) for assessing provider language fluency.

### Study Design

A retrospective cohort design was used to determine whether limited English proficient patients, receiving usual care, had better diabetes-related outcomes over time based on the type and amount of language service they received at primary care visits. To answer this question, we utilized language service data for limited English proficient patients receiving care over 9 months at Cambridge Health Alliance (July 1, 2007–March 31, 2008) and then examined their records for 6 months following the study period to obtain data regarding diabetes outcomes.

### Study Participants

The study population was drawn from patients enrolled in the Cambridge Health Alliance diabetes registry prior to July 1, 2007. The registry is an electronic listing of type II diabetic patients and their clinical information that is used to monitor clinical indicators. Only patients whose preferred language of care – identified at initial registration at Cambridge Health Alliance – was a language other than English were designated as limited English proficient and included in the study. Additional study inclusion criteria included being aged ≥18 years and having at least one primary care visit in both the study period (July 1, 2007 to March 31, 2008) and in the subsequent 6 months. In addition, we eliminated 16 patients who used multiple primary care sites, as we were concerned that they might differ from other patients in ways that would influence our results (for example, they may be less connected to their primary care provider). This was a small number and considered insignificant.

### Dataset Construction

The dataset was constructed using administrative and clinical data from each participant's medical hospital registration and electronic medical records including demographic characteristics (sex, age, preferred language of care), insurance, clinical data (clinical visits, treatment, and outcomes related to type II diabetes), utilization data (Current Procedural Terminology (CPT) and International Classification of Diseases (ICD) codes, procedures, prescriptions, laboratories, and radiologic tests), and administrative data from the interpreter services database (number, length, and modality of interpreter encounters; language of interpreted encounters; and records from vendors used for telephonic interpreting).

### Defining Limited English Proficiency and Language Services

For the purposes of this study, limited English proficient patients were defined as those whose response to preferred language for communication with health care providers was a language other than English. Formal interpreting was defined as a visit with either a face-to-face or telephonic trained medical interpreter. If a provider was listed in the Cambridge Health Alliance provider registry as speaking the same language as the limited English proficient patient, they were considered a language-concordant provider for the purposes of this study. That is, the language of the provider had to match the preferred language of the patient.

### Construction of Language Services Exposure Groups

To examine differences by modality and amount of language service, the percent of primary care visits with 1) formal interpretation services and/or 2) a language-concordant provider was calculated to convey the percent of their visits with language services and the mix of the language services received for each patient at any of their visits in the time period. Any single visit during the study period involving both formal interpretation and a language-concordant provider (N = 153) was coded as a formal interpretation visit because of the high likelihood that the language-concordant provider felt formal interpretation was needed to effectively communicate with the patient.

Each continuous exposure variable (formal interpretation visits and language-concordant provider visits) was subsequently categorized into *three groups based on histograms*. For primary care visits utilizing formal interpretation, histograms suggested three tertiles that allowed for reasonable sample sizes per group: zero (n = 866), 1–49% (n = 288), and 50–100% (n = 303). For patients using language-concordant providers and no formal interpretation, however, the histogram was bimodal with over 60% of the patients receiving zero language-concordant provider contact and approximately 20% receiving 100% of their visits with a language-concordant provider. Therefore, we decided to create the following three categories for language-concordant provider visits: zero (n = 866), 1–99% (n = 269), and 100% (n = 290). This also allowed us to retain a separate language-concordant provider category which is often considered the “gold standard” for communication with limited English proficient patients [32]. Then based upon the cross tabulation of the two categorical variables, patients were assigned to one of seven exposure categories;. Patients with no formal interpreter services and no language-concordant providers; Patients with no formal interpreter services and 1–99% of their visits with language-concordant providers; Patients with no formal interpreter services and 100% of their visits with language-concordant providers; Patients with 1–49% of their visits with formal interpreter services and no visits with language-concordant providers; Patients with 1–49% of their visits with formal interpreter services and 1–99% of their visits with language-concordant providers; Patients with 50–100% of their visits with formal interpreter services and no visits with language- concordant providers; and finally, Patients with 50–100% of their visits with formal interpreter services and1–99% of their visits with language-concordant providers.

### Outcomes

The outcomes of interest included any of the following events during the 6 months following the study period: 1) hospitalizations related to diabetes, 2) emergency department (ED) visits related to diabetes, 3) ED visits not related to diabetes, 4) hospitalizations and/or ED visits related to diabetes (combined), and 5) Hemoglobin (Hgb) A1c level ≥9.0.

Each hospitalization or ED visit was categorized as diabetes-related by examining the first three diagnostic codes (ICD–9) recorded during the event. Diabetes-related ICD-9 codes were identified based on existing literature [36]. The last HgbA1c level recorded during the outcome period was used to construct the HgbA1c outcome variable. The cutoff of ≥9 HgbA1c as poorly controlled diabetes was selected based upon current clinical performance measures for ambulatory care [37].

### Other Variables

Age was grouped into 5 categories (18–39, 40–49, 50–59, 60–69, and 70 and older). Race/ethnicity was categorized as non-Hispanic white, non-Hispanic black, Hispanic, Asian, and other/unknown. Health insurance status was classified as private, public (Medicaid and Medicare), and Free Care/Safety Net/out-of-pocket. Language preferred in medical encounters included Portuguese, Haitian Creole, Spanish, and other/unknown. As a measure of disease complexity, all ICD-9 codes used for each patient during the study period were converted into one of the 30 co-morbidity categories defined by the Elixhauser taxonomy [38]. The patient's total number of co-morbidities was categorized (0–1, 2–3, and 4 or more co-morbidities). The number of primary care visits during the study period was categorized (1–2, 3–4, 5–6, and 7 or more).

### Analysis

The demographic and clinical characteristics (sex, age, race/ethnicity, language, health insurance, number of co-morbidities, number of primary care visits, and HgbA1c) of the overall study population and each language services exposure group were described using frequencies and means based on the patient's last primary care visit during the study period.

To determine whether these characteristics differed by receipt of language services, Chi-square or Fisher's Exact tests were used to compare each exposure group to the reference group (Patients with no formal interpreting services and no language-concordant providers). Patient demographic and clinical characteristics were also tested using Chi-square or Fisher's Exact tests to determine if they were significantly associated with each outcome.

Generalized estimating equation models were developed to examine the relationship between language services group and each binary outcome while controlling for the demographic and clinical characteristics. In final models, language was retained over race/ethnicity as the two were found to be highly correlated. All models included were further adjusted for clustering within primary care site (N = 14). Odds-ratios, 95% confidence intervals, and significant P-values are reported. All analyses were conducted in SAS version 9.1 (SAS Institute, Cary, NC). To adjust for multiple comparisons given that we were looking at five independent outcomes, we used a Bonferoni correction and considered levels of P≤0.01 as statistically significant.

## Results

Prior to July 1, 2007, there were 2,803 limited English proficient patients in the Cambridge Health Alliance diabetes registry. A total of 1,425 limited English proficient patients met all inclusion criteria. Those meeting study inclusion were significantly more likely to be women, <70 years of age, non-Hispanic black and Haitian Creole speakers than those not meeting study criteria.

Demographic and clinical characteristics of the study population are shown in Table 1. The population was racially and linguistically diverse and almost two-thirds had public health insurance. About 20% of patients had poorly controlled diabetes as defined by HgbA1c level ≥9.0.

**Table 1:** Demographic and Clinical Characteristics of Limited English Proficient Population N = 1425.

		Number (Percent)
**Sex**	Female	871 (61.1)
	Male	554 (38.9)
**Age**	18 to 39	85 (6)
	40 to 49	183 (12.8)
	50 to 59	360 (25.3)
	60 to 69	385 (27)
	70 and Over	412 (28.9)
**Race/Ethnicity**	White, Non-Hispanic	399 (28)
	Black, Non-Hispanic	402 (28.2)
	Hispanic	327 (23)
	Asian, Non-Hispanic	64 (4.5)
	Other/Unknown	233 (16.4)
**Language Preferred**	Portuguese	494 (34.7)
	Haitian Creole	391 (27.4)
	Spanish	296 (20.8)
	Other/Unknown	244 (17.1)
**Insurance**	Private	239 (16.8)
	Public	939 (65.9)
	Free Care/Safety Net/Out of Pocket	247 (17.3)
**Co-morbidities**	0 to 1	389 (27.3)
	2 to 3	910 (63.9)
	4 or More	126 (8.8)
	Median Co-morbidities, number (Range)	2 (0–9)
**Primary Care Visits**	1 to 2	345 (24.2)
	3 to 4	514 (36.1)
	5 to 6	283 (19.9)
	7 or More	283 (19.9)
**Hemoglobin A1c Level (n = 1397)** *	<7.0	486 (34.8)
	≥7.0 and <9.0	634 (45.4)
	≥9.0	277 (19.8)

Characteristics based upon last observation during study period.

* Some patients had no documented Hemoglobin A1c.

Thirty-one percent of the limited English proficient patients (445) had no documentation of interpreter use or contact with a language-concordant provider in any of their ambulatory visits. For those who did receive language services (980), 17% (170) of them accessed multiple modalities (formal interpreting, language-concordant provider) across their visits and the majority (499/51%) had at least one visit without any recorded language services.

In bivariate comparisons (Table 2) patients without language services were significantly younger than those in other groups, while most patients who received some language-concordant provider language services or a mix of language-concordant provider with some formal interpreting (Patients with no formal interpreting services and 1–99% of their visits with language- concordant providers; Patients with no formal interpreting services and 100% of their visits with language- concordant providers and Patients with 1–49% of their visits with formal interpreting and 1–99%of their visits with language-concordant providers) were more likely to be white and Hispanic and less likely to be black (except Patients with 50–100% of their visits with formal interpreting services and 1–99% of their visits with language-concordant providers who did not differ by race/ethnicity). Patients whose preferred language was Portuguese were more likely to have received language services, especially a language-concordant provider. Patients who only saw a language-concordant provider (Patients with no formal interpreting services and 1–99% of their visits with language-concordant providers and Patients with no formal interpreting services and 100% of their visits with language-concordant providers) were more likely to speak Spanish and less likely to speak Haitian Creole or other/unknown languages than those receiving no services. Finally, all groups were significantly more likely to have had 4 or more co-morbidities as well as 7 or more primary care visits during the study period than patients receiving no language services.

**Table 2:** Demographic and Clinical Characteristics of Limited English Proficient Population, by Language Services Exposure Group N = 1425.

	Patients with No Formal Interpreter Services and No Language- Concordant Providers	Patients with No Formal Interpreter Services and 1–99% of their visits with Language-Concordant Providers		Patients with No Formal Interpreter Services and 100% of visits with Language-Concordant Providers		Patients with 1–49% of their visits with Formal Interpreter Services and No visits with Language-Concordant Providers		Patients with 1–49% of their visits with Formal Interpreter Services and 1–99% of their visits with Language- Concordant Providers		Patients with 50–100% of their visits with Formal Interpreter Services and No Visits with Language- Concordant Providers		Patients with 50–100% of their visits with Formal Interpreter Services and 1–99% of their visits with Language- Concordant Providers	
	N (%)	N (%)	P*	N (%)	P	N (%)	P	N (%)	P	N (%)	P	N (%)	P
**Sanple**	445 (31.2)	99 (6.9)		290 (20.4)		170 (11.9)		118 (8.3)		251 (17.6)		52 (3.7)	
													
**Sex**			0.03		0.18		0.003		0.004		0.41		0.42
Female	248 (55.7)	67 (67.7)		176 (60.7)		117 (68.8)		83 (70.3)		148 (59)		32 (61.5)	
Male	197 (44.3)	32 (32.3)		114 (39.3)		53 (31.2)		35 (29.7)		103 (41)		20 (38.5)	
**Age**			0.03		0.002		0.07		<0.001		0.04		0.12
18 to 39	30 (6.7)	6 (6.1)		13 (4.5)		13 (7.7)		3 (2.5)		17 (6.8)		3 (5.8)	
40 to 49	80 (18)	10 (10.1)		33 (11.4)		18 (10.6)		8 (6.8)		29 (11.6)		5 (9.6)	
50 to 59	124 (27.9)	21 (21.2)		64 (22.1)		42 (24.7)		27 (22.9)		69 (27.5)		13 (25)	
60 to 69	118 (26.5)	28 (28.3)		90 (31)		47 (27.7)		29 (24.6)		61 (24.3)		12 (23.1)	
70 and Over	93 (20.9)	34 (34.3)		90 (31)		50 (29.4)		51 (43.2)		75 (29.9)		19 (36.5)	
**Race/Ethnicity**			<0.001		<0.001		0.08		<0.001		0.51		0.09
White, Non-Hispanic	84 (18.9)	44 (44.4)		140 (48.3)		19 (11.2)		42 (35.6)		57 (22.7)		13 (25)	
Black, Non-Hispanic	144 (32.4)	3 (3)		26 (9)		71 (41.8)		49 (41.5)		86 (34.3)		23 (44.2)	
Hispanic	83 (18.7)	43 (43.3)		94 (32.4)		28 (16.5)		24 (20.3)		47 (18.7)		8 (15.4)	
Asian, Non-Hispanic	32 (7.2)	0 (0)		8 (2.8)		10 (5.9)		0 (0)		14 (5.6)		0 (0)	
Other/Unknown	102 (22.9)	9 (9.1)		22 (7.6)		42 (24.7)		3 (2.5)		47 (18.7)		8 (15.4)	
**Language Preferred**			<0.001		<0.001		0.08		<0.001		<0.001		<0.001
Portuguese	86 (19.3)	57 (57.6)		167 (57.6)		29 (17.1)		49 (41.5)		85 (33.9)		21 (40.4)	
Haitian Creole	140 (31.5)	1 (1)		25 (8.6)		71 (41.8)		45 (38.1)		86 (34.3)		23 (44.2)	
Spanish	73 (16.4)	40 (40.4)		91 (31.4)		28 (16.5)		22 (18.6)		34 (13.6)		8 (15.4)	
Other/Unknown	146 (32.8)	1 (1)		7 (2.4)		42 (24.7)		2 (1.7)		46 (18.3)		0 (0)	
**Insurance**			0.25		0.85		0.03		0.11		<0.001		0.007
Private	89 (20)	26 (26.3)		63 (21.7)		19 (11.2)		17 (14.4)		21 (8.4)		4 (7.7)	
Public	286 (64.3)	55 (55.6)		182 (62.8)		125 (73.5)		88 (74.6)		171 (68.1)		32 (61.5)	
Free Care/Safety Net/ Pocket	70 (15.7)	18 (18.2)		45 (15.5)		26 (15.3)		13 (11)		59 (23.5)		16 (30.8)	
**Co-morbidities**			<0.001		0.003		<0.001		<0.001		0.03		0.002
0 to 1	144 (32.4)	30 (30.3)		86 (29.7)		37 (21.8)		16 (13.6)		71 (28.3)		5 (9.6)	
2 to 3	285 (64)	52 (52.5)		176 (60.7)		113 (66.5)		81 (68.6)		160 (63.8)		43 (82.7)	
4 or More	16 (3.6)	17 (17.2)		28 (9.7)		209 (11.8)		21 (17.8)		20 (8)		4 (7.7)	
**Primary Care Visits**			<0.001		0.005		<0.001		<0.001		0.001		<0.001
1 to 2	147 (33)	7 (7.1)		123 (42.4)		0 (0)		0 (0)		61 (24.3)		7 (13.5)	
3 to 4	185 (41.6)	34 (34.3)		99 (34.1)		62 (36.5)		27 (22.9)		92 (36.7)		15 (28.9)	
5 to 6	74 (16.6)	34 (34.3)		32 (11)		41 (24.1)		30 (25.4)		58 (23.1)		14 (26.9)	
7 or More	39 (8.8)	24 (24.2)		36 (12.4)		67 (39.4)		61 (51.7)		40 (15.9)		16 (30.8)	
**Hemoglobin A1c** N = 1397 **			0.48		0.06		0.15		0.94		0.26		0.95
<7.0	158 (36.6)	30 (30.3)		109 (38.7)		49 (29.2)		40 (34.8)		81 (32.5)		19 (36.5)	
≥7.0 and <9.0	190 (44)	49 (49.5)		137 (48.6)		77 (45.8)		52 (45.2)		107 (43)		22 (42.3)	
≥9.0	84 (19.4)	20 (20.2)		36 (12.8)		42 (25)		23 (20)		61 (24.5)		11 (21.2)	

* P = P-Value. P-values determined from Chi-square/Fisher's Exact test comparing each language services group to the referent group (Patients with No Formal Interpreter Services/No Language-Concordant Providers).

** Some patients had no documented Hemoglobin A1c.

Language service categories were also significantly related to clinical outcomes (Table 3). In unadjusted analyses, patients who received a mixture of language services (Patients with 1–49% of their visits with formal interpreting services and 1–99% of their visits with language-concordant providers and Patients with 50–100% of their visits with formal interpreting services and 1–99% of their visits with language- concordant providers) were more likely to have experienced a hospitalization or ED visit related to diabetes (over 19.5%/23 and 19.2%/10 respectively) compared to other groups in the outcome period. Patients with 100% of their visits with a language-concordant provider appeared to be least likely to have experienced an ED visit related to diabetes (less than 3%/8). Spanish-speaking patients were more likely than other language groups to have experienced an ED visit related to diabetes. Hospitalizations and/or ED visits related to diabetes were significantly associated with a higher number of co-morbidities, higher numbers of primary care visits, and having public insurance. Non-diabetes related ED visits were not significantly related to language service categories.

**Table 3:** Bivariate Associations between Demographic and Clinical Characteristics and Clinical Outcomes N = 1425.

	Hospitalizations			Emergency Visits						Hospitalizations and/or Emergency Visits			Hemoglobin A1c ≥9.0		
	Related to Diabetes			Related to Diabetes			Not related to Diabetes			Related to Diabetes					
	N	%	P	N	%	P	N	%	P	N	%	P	N	%	P
**Language Services Group**			0.005			0.02			0.29			0.002			0.06
Patients with No Formal Interpreter Services and No Language-Concordant Providers (n = 445)	18	4.0		35	7.9		32	7.2		49	11.0		92	20.7	
Patients with No Formal Interpreter Services and 1–99% of their visits with Language- Concordant Providers (n = 99)	6	6.1		7	7.1		5	5.1		11	11.1		21	21.2	
Patients with No Formal Interpreter Services and 100% of their visits with Language-Concordant Providers (n = 290)	12	4.1		8	2.8		16	5.5		17	5.9		39	13.5	
Patients with 1–49% of their visits with Formal Interpreter Services and No visits with Language-Concordant Providers (n = 170)	13	7.7		8	4.7		17	10.0		19	11.2		37	21.8	
Patients with 1–49% of their visits with Formal Interpreter Services and 1–99% of visits with Language-Concordant Providers (n = 118)	16	13.6		12	10.2		13	11.0		23	19.5		22	18.6	
Patients with 50–100% of their visits with Formal Interpreter Services and No Visits with Language-Concordant Providers (n = 251)	17	6.8		22	8.8		18	7.2		35	13.9		59	23.5	
Patients with 50–100% of their visits with Formal Interpreter Services and 1–99% of their visits with Language- Concordant Providers (n = 52)	5	9.6		6	11.5		2	3.9		10	19.2		14	26.9	
**Sex**			0.05			0.05			0.40			0.01			0.24
Female (n = 871)	62	7.1		69	7.9		67	7.7		115	13.2		165	18.9	
Male (n = 554)	25	4.5		29	5.2		36	6.5		49	8.8		119	21.5	
**Age**			0.02			0.13			0.13			0.15			<0.001
18 to 39 (n = 85)	4	4.7		10	11.8		9	10.6		12	14.1		30	35.3	
40 to 49 (n = 183)	5	2.7		14	7.7		19	10.4		17	9.3		58	31.7	
50 to 59 (n = 360)	19	5.3		20	5.6		29	8.1		36	10.0		77	21.4	
60 to 69 (n = 385)	21	5.5		20	5.2		21	5.5		39	10.1		67	17.4	
70 and Over (n = 412)	38	9.2		34	8.3		25	6.1		60	14.6		52	12.6	
**Race/Ethnicity**			0.73			0.07			0.63			0.27			0.003
White, Non-Hispanic (n = 399)	20	5.0		18	4.5		25	6.3		37	9.3		68	17.0	
Black, Non-Hispanic (n = 402)	26	6.5		30	7.5		28	7.0		49	12.2		107	26.6	
Hispanic (n = 327)	24	7.3		32	9.8		29	8.9		47	14.4		56	17.1	
Asian, Non-Hispanic (n = 64)	3	4.7		3	4.7		6	9.4		6	9.4		10	15.6	
Other/Unknown (n = 233)	14	6.0		15	6.4		15	6.4		25	10.7		43	18.5	
**Language Preferred**			0.26			0.03			0.68			0.04			0.002
Portuguese (n = 494)	26	5.3		25	5.1		32	6.5		47	9.5		85	17.2	
Haitian Creole (n = 391)	24	6.1		30	7.7		28	7.2		47	12.0		104	26.6	
Spanish (n = 296)	25	8.5		30	10.1		26	8.8		47	15.9		55	18.6	
Other/Unknown (n = 244)	12	4.9		13	5.3		17	7.0		23	9.4		40	16.4	
**Insurance**			0.07			0.06			0.34			0.04			0.12
Private (n = 239)	9	3.8		8	3.4		13	5.4		17	7.1		50	20.9	
Public (n = 939)	67	7.1		72	7.7		68	7.2		121	12.9		174	18.5	
Free Care/Safety Net or Out of Pocket (n = 247)	11	4.5		18	7.3		22	8.9		26	10.5		60	24.3	
**Co-morbidities**			<0.001			0.02			0.56			<0.001			0.30
0 to 1 (n = 389)	10	2.6		17	4.4		26	6.7		24	6.2		88	22.6	
2 to 3 (n = 910)	59	6.5		67	7.4		65	7.1		113	12.4		172	18.9	
4 or More (n = 126)	18	14.3		14	11.1		12	9.5		27	21.4		24	19.1	
**Primary Care Visits**			<0.001			0.03			0.03			<0.001			0.21
1 to 2 (n = 345)	11	3.2		15	4.4		20	5.8		24	7.0		63	18.3	
3 to 4 (n = 514)	21	4.1		31	6.0		28	5.5		48	9.3		97	18.9	
5 to 6 (n = 283)	22	7.8		24	8.5		29	10.3		39	13.8		55	19.4	
7 or More (n = 283)	33	11.7		28	9.9		26	9.2		53	18.7		69	24.4	
**Hemoglobin A1c Level** (**n = 1,397**)*			0.95			0.13			0.82			0.16			<0.001
<7.0 (n = 486)	29	6.0		27	5.6		38	7.8		51	10.5		7	1.4	
≥7.0 and <9.0 (n = 634)	38	6.0		43	6.8		44	6.9		69	10.9		98	15.5	
≥9.0 (n = 277)	18	6.5		26	9.4		19	6.9		41	14.8		177	63.9	

Abbreviations: P = P-values determined from Chi-square/Fisher's Exact test * Some patients had no documented Hemoglobin A1c levels.

Number and (Percent) of Sample Experiencing Each Outcome: Hospitalization related to Diabetes = 87 (6.1%); Emergency Visits related to Diabetes = 98 (6.9%); Emergency Visits not related to Diabetes = 103 (7.2%); Hospitalizations and/or Emergency Visits Related to Diabetes = 164 (11.5%); Hemoglobin A1c ≥9.0 = 284 (19.9%).

Results of the multivariate models, adjusting for age, gender, language, insurance, co-morbidities, primary care visits, HgbA1c level, and main primary care site, are seen in Table 4. (We ran models with and without adjustment for primary care visits, recognizing that number of primary care visits could be related either to severity of disease or to issues with access to language services and thus minimize our findings. However, none of our outcomes substantially differed between the two models.) Patients who received 100% of their visits with language-concordant providers were less likely to have an ED visit related to diabetes or poorly controlled diabetes compared to patients receiving no language services. The odds of an ED visit related to diabetes was also 63% less for patients with 1–49% of their visits with formal interpretation and no visits with a language-concordant provider compared to the reference group. Again, in multivariate models, non-diabetes related ED visits were not significantly related to language service categories or other variables.

**Table 4:** Multivariate Models of Clinical Outcomes (N = 1397).

	Hospitalizations			Emergency Visits						Hospitalizations and/or Emergency Visits			Hemoglobin A1c ≥9.0		
	Related to Diabetes			Related to Diabetes			Not related to Diabetes			Related to Diabetes					
	OR	95% CI	P	OR	95% CI	P	OR	95% CI	P	OR	95% CI	P	OR	95% CI	P
**Language Services Group**															
Patients with No Formal Interpreter Services and No Language-Concordant Providers (n = 445)	1.00			1.00			1.00			1.00			1.00		
Patients with No Formal Interpreter Services and 1–99% of their visits with Language- Concordant Providers (n = 99)	0.82	0.34–2.01	0.67	0.53	0.20–1.42	0.21	0.58	0.27–1.28	0.18	0.55	0.23–1.29	0.17	1.34	0.71–2.52	0.37
Patients with No Formal Interpreter Services and 100% of their visits with Language-Concordant Providers (n = 290)	0.79	0.41–1.50	0.47	**0.28**	**0.17–0.46**	<**0.001**	0.77	0.29–2.00	0.59	**0.39**	**0.28–0.55**	<**0.001**	0.94	0.63–1.39	0.74
Patients with 1-49% of their visits with Formal Interpreter Services and No visits with Language-Concordant Providers (n = 170)	1.21	0.56–2.60	0.63	**0.37**	**0.20–0.69**	<**0.001**	1.24	0.56–2.78	0.6	0.63	0.39–1.04	0.07	0.82	0.36–1.84	0.63
Patients with 1-49% of their visits with Formal Interpreter Services and 1–99% of visits with Language- Concordant Providers (n = 118)	1.90	1.09–3.34	0.02	0.79	0.35–1.81	0.58	1.36	0.66–2.80	0.41	1.06	0.60–1.89	0.84	0.96	0.45–2.01	0.91
Patients with 50–100% of their visits with Formal Interpreter Services and No Visits with Language- Concordant Providers (n = 251)	1.45	0.65–3.23	0.37	0.94	0.50–1.76	0.84	0.92	0.47–1.82	0.81	1.09	0.63–1.86	0.76	1.08	0.69–1.69	0.73
Patients with 50–100% of their visits with Formal Interpreter Services and 1–99% of their visits with Language- Concordant Providers (n = 52)	1.68	0.69–4.12	0.25	1.08	0.32–3.57	0.90	0.43	0.10–1.82	0.25	1.31	0.57–3.02	0.52	1.65	0.98–2.78	0.06
Sex															
Female	1.00			1.00			1.00			1.00			1.00		
Male	0.69	0.41–1.15	0.15	0.65	0.43–0.98	0.04	0.82	0.49–1.37	0.45	0.67	0.46–0.97	0.03	1.06	0.78–1.45	0.71
Age															
18 to 39	1.00			1.00			1.00			1.00			1.00		
40 to 49	0.57	0.19–1.69	0.31	0.65	0.26–1.67	0.37	1.00	0.36–2.80	1.0	0.64	0.28–1.45	0.28	1.56	0.89–2.72	0.12
50 to 59	0.94	0.31–2.82	0.91	0.42	0.17–1.02	0.06	0.71	0.30–1.67	0.43	0.61	0.26–1.47	0.27	0.62	0.32–1.18	0.14
60 to 69	1.02	0.39–2.65	0.97	0.42	0.17–1.03	0.06	0.46	0.22–0.97	0.04	0.67	0.32–1.41	0.29	0.57	0.30–1.06	0.08
70 and Over	1.56	0.72–3.37	0.26	0.61	0.27–1.36	0.23	0.47	0.18–1.21	0.12	0.89	0.47–1.70	0.73	**0.35**	**0.19–0.64**	**<0.001**
**Language Preferred**															
Portuguese	1.00			1.00			1.00			1.00			1.00		
Haitian Creole	0.96	0.52–1.76	0.90	1.15	0.74–1.80	0.53	1.02	0.58–1.81	0.93	0.95	0.58–1.55	0.83	**1.66**	**1.38–1.99**	<**0.001**
Spanish	2.09	1.02–4.28	0.04	**2.30**	**1.33–3.97**	<**0.001**	1.25	0.84–1.87	0.27	**2.07**	**1.23–3.47**	<**0.001**	0.87	0.63–1.19	0.37
Other/Unknown	1.11	0.60–2.08	0.74	0.91	0.40–2.05	0.81	0.93	0.36–2.42	0.89	0.92	0.49–1.75	0.80	1.14	0.74–1.74	0.55
**Insurance**															
Private	1.00			1.00			1.00			1.00			1.00		
Public	1.08	0.42–2.77	0.87	1.85	0.94–3.64	0.08	1.46	0.84–2.54	0.18	1.30	0.75–2.26	0.35	0.99	0.59–1.67	0.97
Free Care/Safety Net/Out of Pocket	1.05	0.41–2.72	0.92	**1.89**	**1.16–3.07**	**0.01**	1.61	0.67–3.87	0.29	1.28	0.73–2.24	0.39	1.14	0.66–1.99	0.64
**Co-morbidities**															
0 to 1	1.00			1.00			1.00			1.00			1.00		
2 to 3	1.95	0.88–4.33	0.10	1.64	0.94–2.85	0.08	1.08	0.71–1.63	0.72	1.88	1.12–3.15	0.02	0.94	0.67–1.33	0.72
4 or More	**3.59**	**1.35–9.50**	**0.01**	2.48	1.07–5.74	0.03	1.40	0.63–3.11	0.41	**3.14**	**1.42–6.95**	**0.005**	1.09	0.79–1.50	0.60
**Primary Care Visits**															
1 to 2	1.00			1.00			1.00			1.00			1.00		
3 to 4	1.03	0.41–2.60	0.95	1.27	0.88–1.83	0.20	0.86	0.53–1.39	0.55	1.18	0.77–1.79	0.45	1.08	0.55–2.12	0.82
5 to 6	1.69	0.87–3.23	0.12	1.67	1.01–2.77	0.05	1.74	1.03–2.95	0.04	1.58	1.00–2.51	0.05	0.92	0.56–1.51	0.73
7 or More	2.03	0.95–4.32	0.07	1.83	1.00–3.32	0.05	1.38	0.62–3.06	0.43	**1.97**	**1.20–3.21**	**0.007**	1.29	0.52–3.22	0.59
**Hemoglobin A1c**															
<7.0	1.00			1.00			1.00			1.00			1.00		
≥7.0 and <9.0	0.91	0.55–1.50	0.71	1.24	0.72–2.11	0.44	0.86	0.65–1.13	0.28	1.02	0.62–1.67	0.94	**10.72**	**5.48–20.97**	**<0.001**
≥9.0	1.01	0.65–1.57	0.95	1.53	0.78–3.00	0.22	0.73	0.50–1.07	0.10	1.38	0.87–2.18	0.18	**101.18**	**54.03–189.48**	**<0.001**

Abbreviations: OR Odds Ratio; CI Confidence Interval.

OR, 95% CI, and P-values determined from GEE models containing all covariates shown unless otherwise noted (-); all models were further adjusted for site of primary care.

## Discussion

In this study we found that type of language services received by diabetic patients was significantly related to relevant utilization outcomes. Of particular note, patients seeing language-concordant providers at 100% of their primary care visits were least likely to have diabetes-related hospitalization and emergency visits.

### Use of Language Services

Unexpectedly, almost a third of the patients did not receive any language services or visits with a language-concordant provider and many of the remaining patients had at least one visit without language services. One explanation for this finding is that patients often use “ad hoc” interpreting, [39], [40] which has been defined as having an untrained family member, friend or clinic employee interpreting during the visit. It is also possible that use of interpreters was somehow related to the nature of the visit itself and may have been influenced by factors including the patient's acuity, the level of complexity (duration, number of issues addressed, etc.) or the need for intensive patient education. For example, the limited English proficient patients who did not receive language services tended to be younger, to have fewer co-morbidities and to have fewer primary care visits, suggesting that they were relatively healthier and that more seriously ill patients were more likely to obtain language services. Similarly, those with lower usage of language services might also have fewer acute medical needs and thus be less likely to utilize the ED. These characteristics might play a significant role in determining when limited English proficient patients receive interpreting services, both from the perspective of the patient as well as that of the provider [41].

It is also possible that while the data recorded in the hospital registration system indicates that patients had a non-English language of care – and were therefore defined as limited English proficient in our study – some may have spoken enough English to influence their or their providers' request for formal interpretation. Unfortunately, given available information, it is impossible to determine which of these explanations is most likely. Further research is needed to understand how decisions are made to access language services and to determine how communication was handled in these visits without language services.

There may also be specific cultural differences in some groups that account for their access to language services. For example, Portuguese-speaking patients received the most language services suggesting that they may be somehow more active in their own care or in seeking out services. This could be contributing to their lower rate of diabetes-related ED visits.

In general, limited English proficient patients utilized varied modes of language services across their visits and even at the same visit. This made it difficult to determine the amount of services they received and the impact of that amount on outcomes. As we try to determine the evidence base for any one particular modality of language service we must realize that the use of a single modality across all visits is unlikely in actual practice, especially in light of variability in interpreter availability, language-concordant provider availability, and complexity of visits, as well as the dynamic nature of patients' levels of English proficiency.

### Impact on Outcomes

Patients with 100% language-concordant provider visits were significantly less likely to have an ED visit related to their diabetes and to have either a hospitalization or ED visit related to diabetes compared to others, even when controlling for demographic and clinical characteristics. While the group was observed to have fewer co-morbidities and lower HgbA1c levels in bivariate analyses, these differences were not significant in multivariate models controlling for demographic and clinical characteristics. Thus, it is possible that language concordance may enhance the communication between patient and provider and lead to improved health outcomes [20], [42], [43], [44]. Fernandez et al. reported that glycemic control was enhanced by having a language-concordant provider in a Spanish-speaking population whereas disparities in glycemic control persisted for limited English proficient patients who had language discordant providers [28]. More research is needed to understand how provider language fluency impacts health outcomes particularly given that language proficiency may vary among providers [43].

Additional demographic factors that were predictive of hospitalization and ED visits included speaking Spanish, having more than 4 co-morbidities, and having more than one primary care visit. The number of co-morbidities have been related to increased risk for hospitalization and ED in other studies [45] and the number of primary care visits is likely to be related to increased patient need. In addition, several studies have noted higher utilization of ED visits and hospitalization by Latino diabetics compared to non-Latinos [43], [46] but this may differ significantly by state [47]. Our results suggest that these groups may have the most to benefit from language-concordant providers. Further research is needed to ascertain whether this is unique to the CHA population or generalizable elsewhere.

### Limitations

This study has a number of limitations. First, it was conducted in one urban public hospital system serving a diverse and underserved population and findings from such a setting may not be generalizable to other similar populations. In addition, it is possible that we were unable to capture all the visits that these patients had with medical providers since some may have occurred outside of the CHA system. We recognize that since the variables of interest – limited English proficiency and language concordant providers – were based on self-report rather than on objective measures of fluency, it is possible that we either over or underestimated patients' and providers' communication skills. In addition, limited English proficiency was defined based on the answer to preferred language of care and not on a secondary question about English fluency as outlined by Karliner et al [48]. Thus, individuals may have been categorized as limited English proficient by our criteria, but may have had sufficient English proficiency to communicate with their provider during visits. This might have contributed to the finding that some limited English proficient patients did not receive any language services. However, the findings about language concordant providers suggest that our categories were valid and, given that this is a study of a real world health care system, we did not have information necessary to further refine our definitions.

In addition, given the way our language categories were structured with the combining of two types of language assistance, it is possible that we may have underestimated the impact of language services. However, given the tremendous variability in the amount of interpreter services received by individual patients within the sample and the fact that the majority had received multiple modalities of those services, we followed a procedure to give us both equal-sized groups and logical cut points for analysis while preserving the category of language-concordant provider.

We also recognize that patient assignment to a language-concordant provider was dependent on request and availability. It is therefore possible that the language-concordant provider group was vulnerable to some selection bias; however it is unlikely that a patient's request or language-concordant provider availability would differ based on future clinical outcomes. There is also a possibility that preferred language interacted with having a language-concordant provider given that language was associated with the exposure of interest (language services group) as shown in table 3. This may have been due in part to the fact that the ratio of language-concordant providers to language group was different across the languages. Since language met the definition of confounder we controlled for it in the final multivariate models, as we were unable to formally test for interactions between key covariates (such as language) and language services received in our models due to small cell counts.

Other potential confounders associated with patient outcomes [49], including diabetes duration, years of education, and language-concordant provider language proficiency (this was self-reported and not tested), were unavailable and additional research is needed to better understand the contribution of these factors. Despite these limitations, this study is one of the first to examine the impact of amount and type of language services received on diabetes outcomes.

### Conclusions/Recommendations

While this study suggests that language-concordant providers may help reduce health care utilization for limited English proficient patients, it is unlikely that health care systems will ever be able to provide enough language-concordant providers to meet demand. Yet, to insure that limited English proficient patients receive high quality care, multiple strategies are needed to increase the availability of language-concordant providers including recruiting and retaining more bilingual individuals to the health care professions, as well as providing testing and training to build the language capacities of bilingual primary care providers. In the meantime, we need to assess the unique needs of our patients to ensure that the highest risk patients receive the most appropriate language services. Subsequent to this study, Cambridge Health Alliance implemented a new set of questions in their electronic medical record requiring providers to document how they met the language needs of the patient at each visit. Strategies such as this will improve our ability to better understand when and how language services are utilized. The challenges inherent in providing services to a diversifying population deserve further study to determine the best policy and practice strategies to achieve this goal.
